# New Appliances and Things Medical

**Published:** 1905-03-04

**Authors:** 


### NEW APPLIANCES AND THINGS MEDICAL.

[We shall be glad to receive at our Office, 28 & 29 Southampton Street, Strand, London, W.C., from the manufacturers, specimens of all new preparations and appliances which may be brought out from time to time.]

### MAGGI'S ESSENCE (AUX FINES HERBES).

(Cosenza and Co., London).

Maggi's essences and meat extracts are well-known on the continent, but not so well-known in this country. The one before us is the flavouring essence of fine herbs; they also make a truffle flavouring essence. These extracts are distinct from this firm's meat extracts, which are used for making bouillons, etc., are simply flavouring agents and have no value as nutrients. The importance of flavour as an adjuvant to digestion requires, however, no emphasis. If used according to the directions, the essence imparts a pleasant and piquant flavour to hashes, soups, and other dishes. It consists chiefly of amido-bodies (5·73 per cent.) and contains about 1 per cent. of peptones and 22·39 per cent. of ash.

### MALTINE WITH CREOSOTE.

(Maltine Manufacturing Company, Limited, London.)

This preparation consists of a carefully prepared extract of malt, free from alcohol, and also containing an active diastase. The essential feature of malt extracts, and one which distinguishes them from other artificial foods, is that they contain a relatively high proportion of dextrin or soluble starch and maltose, carbohydrate food stuffs of value, together with an active ferment, diastase, which is capable of assisting in the digestion of the ordinary starch of the food. The creosote in this preparation is small in amount, each tablespoonful containing two minims of pure creosote, or little more than the minimal pharmacopœial dose. This creosote acts mainly, and efficiently, as a gastric antiseptic, inhibiting gastric fermentation and consequent distention. This statement is made, as it might be assumed that the creosote would act as a respiratory antiseptic, and hence have a curative value in pulmonary tuberculosis; if this action is desired additional creosote must be given, and can be administered with the maltine.

### QUAKER OATS.

(American Cereal Company, Limited, 11 Finsbury Square.)

A recognised obstacle to the more general use of oatmeal in its usual form is the time required in the process of cooking. A food, therefore, which like Quaker Oats has all the nutritive value of the best quality of oatmeal, and can be rapidly prepared is a distinct advantage, and will find a place alike on the breakfast table and in the nursery and the sick-room. Our personal experience is strongly in support of this preparation.

### LYSOL.

(Schülke and Mayr, 9 & 10 St. Mary-at-Hill, E.C.)

This is a liquid preparation containing a high percentage of cresylic acid, and hence possesses powerful disinfectant and antiseptic properties. It is freely soluble in water, and the solution has a peculiar detergent quality which renders it particularly useful in cleansing such wounds and injuries as are common in general casualty work, and in disinfecting the surgeon's hands previous to operating. Lysol is pre-eminently the antiseptic for obstetrical work.

